# A Knee Size-Independent Parameter for Malalignment of the Distal Patellofemoral Joint in Children

**DOI:** 10.1155/2019/3496936

**Published:** 2019-09-15

**Authors:** Ferdinand Wagner, Günther Maderbacher, Jan Matussek, Boris M. Holzapfel, Birgit Kammer, Jochen Hubertus, Sven Anders, Sebastian Winkler, Joachim Grifka, Armin Keshmiri

**Affiliations:** ^1^Institute of Health and Biomedical Innovation, Queensland University of Technology, 60 Musk Ave, QLD 4059, Brisbane, Australia; ^2^Department of Orthopaedic Surgery, Ludwig-Maximilians-University, Marchioninistrasse 15, 81337 Munich, Germany; ^3^Dr. von Hauner Children's Hospital, Ludwig-Maximilians University of Munich, Lindwurmstrasse 4, 80337 Munich, Germany; ^4^Department of Orthopaedic Surgery for the University of Regensburg at the Asklepios Clinic Bad Abbach, Kaiser-Karl-V. Allee 3, 93077 Bad Abbach, Germany; ^5^Orthopedic Center for Musculoskeletal Research, University of Wuerzburg, Koenig-Ludwig-Haus, Brettreichstr. 11, 97074 Wuerzburg, Germany; ^6^Pediatric Radiology, Department of Radiology, Dr. von Hauner Children's Hospital, Ludwig-Maximilians-University, Lindwurmstrasse 4, 80337 Munich, Germany

## Abstract

**Introduction:**

Patellar instability (PI) is a common finding in children. Current parameters describing patellofemoral joint alignment do not account for knee size. Additionally, most parameters utilize joint-crossing tibiofemoral landmarks and are prone to errors. The aim of the present study was to develop a knee size-independent parameter that is suitable for pediatric or small knees and determines the malpositioning of the distal patellar tendon insertion solely utilizing tibial landmarks.

**Methods:**

Sixty-one pediatric knees were included in the study. The tibial tubercle posterior cruciate ligament distance (TTPCL) was measured via magnetic resonance imaging (MRI). The tibial head diameter (THD) was utilized as a parameter for knee size. An index was calculated for the TTPCL and THD (TTPCL/THD). One-hundred adult knees were analyzed to correlate the data with a normalized cohort.

**Results:**

The THD was significantly lower in healthy females than in males (69.3 mm ± 0.8 mm vs. 79.1 mm ± 0.7 mm; *p* < 0.001) and therefore was chosen to serve as a knee size parameter. However, no gender differences were found for the TTPCL/THD index in the healthy adult study cohort. The TTPCL/THD was significantly higher in adult PI patients than in the control group (0.301 ± 0.007 vs. 0.270 ± 0.007; *p*=0.005). This finding was repeated in the PI group when the pediatric cohort was analyzed (0.316 ± 0.008 vs. 0.288 ± 0.010; *p*=0.033).

**Conclusion:**

The TTPCL/THD index represents a novel knee size-independent measure describing malpositioning of the distal patellar tendon insertion determined solely by tibial landmarks.

## 1. Introduction

Symptoms of patellar instability (PI) usually occur in childhood and adolescence and therefore pose a common problem in the pediatric population [1]. Extensive clinical experience and detailed stepwise analysis of multiple factors, such as dysplasia of the trochlea and patella, leg axis, and rotational alignment, are crucial to understand the pathology of individual patients. A critical point for therapeutic decision making in PI is the location of the distal insertion of the patellar ligament [2, 3].

A pathologic lateralized tibial tubercle often causes PI and patellar dislocation and is commonly addressed by medializing osteotomies, as described by Elmslie–Trillat, in the mature skeletal system and by soft tissue reconstruction, such as medial patellofemoral ligament (MPFL) plasty and the Roux–Goldthwait procedure, in children [4–7].

A frequently utilized parameter indicating the need for distal realignment is the tibial tubercle-trochlear groove distance (TTTG) [8]. This value indicates a mediolateral mismatch of the center of the femoral trochlear groove and the insertion of the distal patellar tendon as determined in the transversal plane generated via computed tomography (CT) or magnetic resonance imaging (MRI) [8–13]. A value of more than 20 mm is usually considered pathological in CT scans. Regularly, smaller values were described for MRI measurements. In recent studies, three major problems have been raised concerning this parameter. First, different positioning of the joint during imaging results in inconsistent values because the landmarks used to measure the TTTG are located across the joint line at the femur and the tibia [14]. Second, the TTTG reflects the absolute value and therefore does not consider differences in overall knee size [10]. Knee size has been shown not only to be different between men and women but also to vary with age [10, 15]. Third, only 56% of the patients with PI present with a pathological TTTG [16]. Recently, Hingelbaum et al. described a knee size-adjusted TTTG index that includes the tibial tubercle-femoral trochlear entrance (TTTE) distance as a knee size-independent parameter measured in the longitudinal plane by MRI [10]. However, the concern of incorrect joint positioning during imaging is also relevant for this parameter.

Seitlinger et al. recently introduced a novel parameter solely utilizing tibial landmarks [17]. The mediolateral distance between the tibial tubercle (TT) and the medial border of the posterior cruciate ligament (PCL) describes the true lateralization of the distal insertion of the patellar tendon [14]. Other authors evaluated this new measure (TTPCL) and proposed pathological values as indicators for the Elmslie–Trillat procedure in the case of adult PI patients [14, 18].

However, the TTPCL does not consider the knee size, and therefore its applicability for children or small knees is questionable. The aim of the presented study was to describe a knee size-independent measure for pathologic lateral malpositioning of the tibial tubercle by determining the ratio between the TTPCL and the maximal tibial head diameter (THD). This TTPCL/THD index might be used as an additional tool in surgical decision-making.

## 2. Methods

### 2.1. Patients

One-hundred MRI scans of knees from adults and 61 knee MRI scans from children were analyzed retrospectively. The presence of open epiphyseal growth plates, and thus the inclusion of each individual patient in the pediatric study cohort, was verified by X-ray examinations. Patients with a history of chronic knee pain without trauma who presented PI in the subsequent clinical examination were included in the PI group. PI was defined as one or more events of patellar dislocation and/or a positive apprehension sign from 0° to 90° of flexion. Patients undergoing MRI because of acute knee pain due to trauma served as a control cohort [15]. These patients had no history of chronic knee pain, retropatellar cartilage defects, or surgery addressing the patellofemoral joint. Standard MRI scans were acquired as a routine procedure in several outpatient radiology clinics.

### 2.2. Measurements

MRI scans were blinded, and three different parameters were assessed by two independent orthopedic surgeons (F. W. and G. M.) utilizing the transversal planes of the T2 sequence. IMPAX Xerox 2014 software (Agfa Health Care, Mortsel, Belgium) was used for the measurements.

The TTTG was defined as the mediolateral distance between the midpoint of the insertion of the patellar tendon and the trochlear groove as described by Goutallier et al. [8]. This distance was measured parallel to the dorsal femoral condylar line.

**Figure 1:**
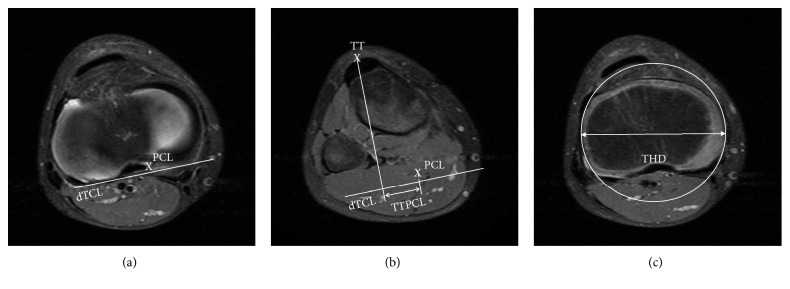
Measurement technique for the TTPCL/THD. Representative T2 MRI sequences acquired from a healthy 9-year-old boy. The mediolateral tibial tubercle-posterior cruciate ligament (TTPCL) distance was measured from (a) the medial border of the tibial insertion of the posterior cruciate ligament (PCL) and (b) the midpoint of the tibial tubercle (TT). The distance was measured parallel to the dorsal tibial condylar line (dTCL). The bony margins in the MRI scans were utilized except when measuring the tibial head diameter (THD).To determine the THD, the transversal plane with the maximum diameter of the tibial head was identified by the examiner (c).

The mediolateral TTPCL distance was measured from the midpoint of the tibial tubercle and the medial border of the posterior cruciate ligament (PCL) as proposed by Seitlinger et al. [17]. The distance was measured parallel to the dorsal tibia condylar line (Figures 1(a) and 1(b)).

For all the measurements, the bony margins in the MRI scans were used, except when measuring the maximal THD. The THD was defined as the proximal part of the tibia with maximal diameter (Figure 1(c)). To determine the THD, the transverse plane with the maximum diameter of the proximal tibial head was identified by the examiner. The outer cartilaginous margin of the tibial head was used when analyzing MRI scans of children as they can be easily determined in the T2 MRI sequence.

### 2.3. Statistical Analysis

Retrospective data acquisition and analysis were approved by the ethics committee of the University of Regensburg (Approval No.: 6-104-0131) and performed according to the Declaration of Helsinki. Inter- and intraobserver variabilities were assessed via intraclass coefficient (ICC) analysis for TTPCL, TTTG, THD, and TTPCL/THD. All parameters were measured twice by both orthopedic surgeons on two separate days. The mean of these parameters for every patient was utilized for consecutive calculations in order to determine significant differences between groups. The results were expressed as the mean per group ± the standard error of the mean (±SEM). The Mann–Whitney *U* test was performed using SPSS (IBM, Ver. 20) to determine statistical significance between groups.

## 3. Results

### 3.1. Patient Characteristics

Forty-two of 100 adult patients suffered from PI, while 58 patients were included in the healthy control group (see Table 1 for detailed patient characteristics). In the second step, 61 knee MRI scans from patients with open epiphyseal growth plates were analyzed. Of the 61 pediatric patients, 32 knees exhibited PI and the remaining knees were assessed as control knees. The mean ages were 12.3 years old (±0.4) in the pediatric PI group and 13.3 years old (±0.3) in the control group (*p*=0.230).

### 3.2. TTPCL, TTTG, and TTPCL/THD

**Figure 2:**
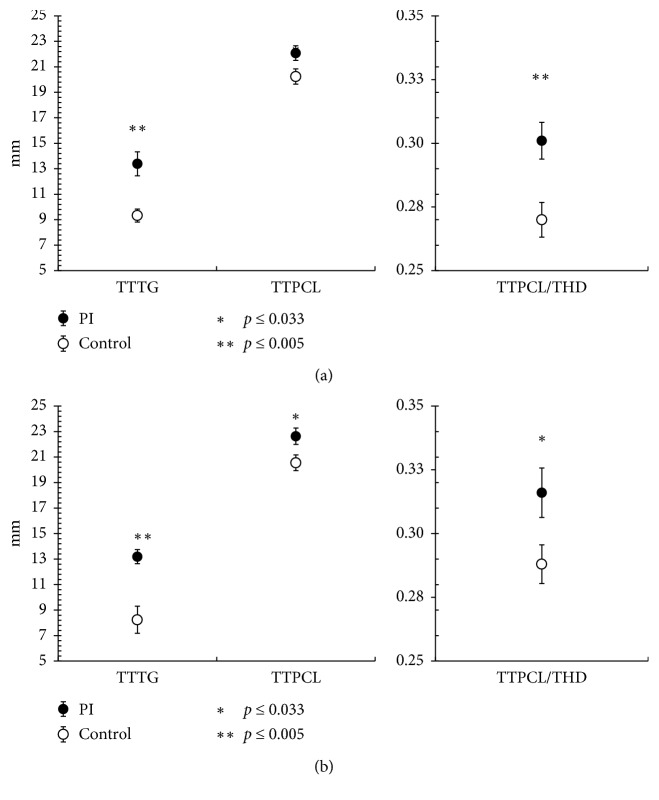
Graphs illustrating the TTTG, TTPCL, and TTPCL/THD for the control and patellar instability (PI) groups for adults (a) and children (b). The results are expressed as the mean values of the group ± the standard error of the mean (SEM).

In adult patients, the TTTG and TTPCL were significantly higher in the PI group (TTTG: 13.4 mm ± 0.94 mm vs. 9.3 mm ± 0.50 mm; *p* ≤ 0.001; TTPCL: 22.1 mm ± 0.57 mm vs. 20.2 mm ± 0.60 mm; *p*=0.031; Table 2 and Figure 2(a)). No difference was observed between the PI and controls for the mean THD when all adult knees were compared. However, our analysis found that the TTPCL/THD index was significantly higher in the adult PI group than in the adult control group (0.301 ± 0.007 vs. 0.270 ± 0.007; *p*=0.005). Accordingly, similar results were found in the pediatric patient population (Table 2 and Figure 2(b)).

### 3.3. Knee Size-Dependent Differences

**Figure 3:**
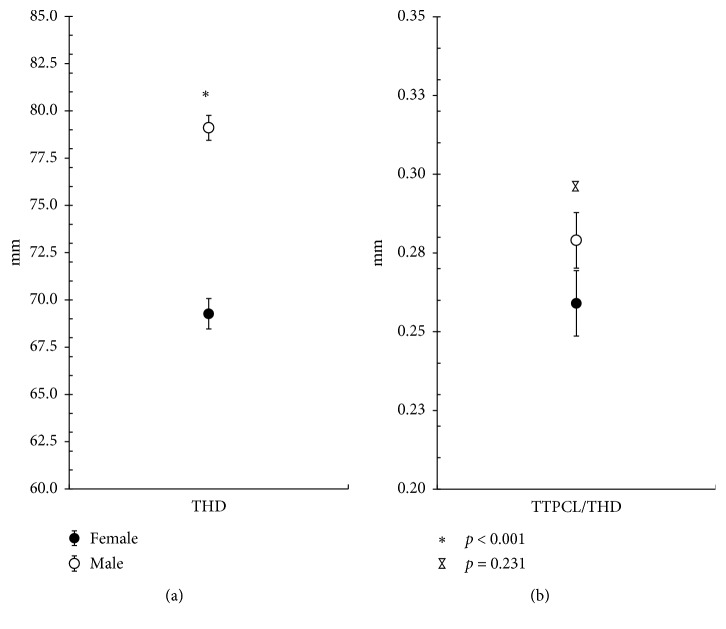
Graphs illustrating the THD and TTPCL/THD for healthy male and female adults. The results are expressed as the mean values of the group ± SEM.

The TTPCL differed significantly between healthy male and female participants (*p* < 0.001; Table 3). As the mean THD was also approximately 10 mm smaller in adult women than in men (69.3 mm ± 0.8 mm vs. 79.1 mm ± 0.7 mm; *p* < 0.001; Table 3 and Figure 3), we regarded both parameters to be knee size-dependent. Therefore, we divided our healthy adult patient cohort into male and female groups to determine whether the TTPCL/THD index is gender- independent and therefore knee size-independent. Consequently, no difference in the TTPCL/THD index was found between the sexes in the healthy adult population (Table 3 and Figure 3).

### 3.4. TTPCL/THD in Children

Because no difference was found in the TTPCL/THD between sexes in adults and, more specifically between knee sizes, we regarded this value as knee size-independent. Therefore, we calculated the TTPCL/THD index for the pediatric study population. We found significantly higher values again in the PI group than that in the pediatric control group (0.316 ± 0.008 vs. 0.288 ± 0.010; *p*=0.033; Table 2 and Figure 2(b)).

### 3.5. Inter- and Intraobserver Correlation

Good to excellent inter- and intraobserver correlations were found between the measurements. The ICC was >0.900 for all parameters when the intraobserver variability was analyzed. The interobserver variability values were ≥0.950 for both the TTPCL/THD and THD. We calculated an interobserver variability of 0.888 for the TTTG and 0.711 for the TTPCL.

## 4. Discussion

Pathologies of the patellofemoral joint leading to PI with consecutive anterior knee pain or patellar dislocation are multifactorial and therefore complex [2]. Although a broad variety of clinical and radiological measures seem to assist in clinical decision-making, safe algorithms have not been established [1, 14, 15]. A major concern in pediatric orthopedics is that many parameters are established in the adult patient population but are not normalized for different joint sizes and consequently are not suitable for immature patients [10, 15]. The TTTG, which is expressed as an absolute value in millimeters, does not consider the fact that a lower TTTG value in a smaller knee is considered abnormal [10]. For that purpose, Hingelbaum et al. have recently described a TTTG Index, measuring the TTTG as well as the distance from the tibial tubercle to the deepest point of the chondral entrance of the trochlea (TTTE) as a parameter for knee size [10]. Studies confirming the reliability of this parameter are missing to date. Others like Graf et al. recently highlighted the quantification of the q vector and the TTTG angle in order to appropriately address TTTG in surgical decision-making [19, 20]. To our knowledge, no attempts have been made to implement age-dependent percentiles for the TTTG.

Recently, a novel parameter was described by Seitlinger et al. that eliminated the limitation of joint line-crossing landmarks as is reported for the TTTG [17, 21]. The TTPCL solely utilizes tibial landmarks and describes the pathological lateralization of the distal insertion of the patellar tendon at the tibial tubercle [17]. However, joint size is still not considered with this measure [14].

Therefore, we designed our study to include a measure of joint size, such as the THD, when analyzing the TTPCL [22]. In analogy to Hingelbaum et al., we performed a stepwise analysis in order to determine, if our parameter is size independent [10]. In a first step, we found that the TTPCL/THD ratio is significantly higher in adult PI patients than in the healthy study population, and we regarded this parameter as a valuable measure for the lateralization of the tibial tubercle. The fact that previously established parameters such as the TTTG and TTPCL were also higher in the PI group confirmed that we analyzed adequate study cohorts that reflect the disease. In a second step, we showed that the THD is a measure of knee size, as it was significantly smaller in women than in men. The TTPCL was also significantly smaller in women than in men. However, no difference was found in the TTPCL/THD index between healthy male and female study participants, indicating that including the THD into the TTPCL parameter eliminates knee size-dependent differences. We also found no difference in the TTPCL/THD index between healthy boys and girls. Nevertheless, a significant difference was found between children with and without PI. Therefore, we also concluded that this measure is a novel knee size-independent parameter for the true lateralization of the tibial tubercle that is applicable in the growing and maturing musculoskeletal system. The finding that healthy adult women have a significantly smaller TTPCL and a smaller THD than men also reflects the importance of the knee size component in adults for this specific parameter.

The mean TTTG was 13.2 mm in our pediatric PI group and only 13.4 mm in the adult group. Both are near the recommended normal position of the tibial tubercle as proposed by Dejour et al. [16] and lower than the pathologic values, which are generally described in the literature to be higher than 15 or 20 mm in CT scans [3, 16]. A study by Camp et al. showed that measuring TTTG by MRI modalities is generally lower than when determined via CT scans (16.9 mm in CT scans versus 14.7 mm in MRI scans in a PI group of 59 knees) [23]. In a subgroup of their patients presenting with a TTTG >20 mm in CT scans (mean 22.5 mm), the mean TTTG distance was only 18.7 mm in MRIs. This resulted in a mean difference of 3.8 mm between both imaging modalities (*p* < 0.001).

The described TTPCL/THD index utilizes only tibial landmarks and therefore is independent of certain modalities during imaging such as joint positioning. This is of particular value as significant variations in the current standard parameter, namely, the TTTG, can be generated by variations in positioning of the joint during MRI or CT examinations [14]. Dietrich et al. described a high variability in TTTG values in healthy volunteers depending on the knee positioning during MRI (ranging from 15.1 mm in full knee extension and 8.1 mm in 30° flexion) [24, 25]. Nevertheless, our TTTG values measured for the pediatric and adult PI cohort are out of the usually recommended cutoff for surgical intervention [26]. As we found no significant difference in TTTG between pediatric and adult patients, our results cannot fully refute TTTG as a nonapplicable parameter for pediatric PI patients. Additionally, our data found differences in value ranges (SEM and min-max) between groups opening up the question if our cohorts are representative for the investigated question. However, we did not want to specify our PI cohort on single radiological parameters and used clinical measures as PI is a multifactorial condition [3]. PI was defined as one or more events of patellar dislocation and/or chronic anterior knee pain with a positive apprehension sign from 0° to 90° of flexion. Patients undergoing MRI because of acute knee pain due to trauma served as a control cohort [15]. Another limitation of our retrospective study is that the pediatric patient cohort was rather small.

We used MRI to evaluate our novel index for two reasons. In contrast to CT, this imaging modality is free of radiation and is therefore favorable for children [27]. Additionally, cartilaginous landmarks can be determined easily by MRI [9, 28], which is especially helpful when measuring the true maximal THD in children.

We found good to excellent intra- and interobserver correlation for all parameters. This corresponds with other reports [14, 17]. Although the ICC for the TTPCL was only fair—with a value of 0.711—the results were comparable to those published by Seitlinger et al. [17]. Additionally, other authors have previously reported values >0.900 for the TTPCL. More importantly, we found excellent values when calculating the TTPCL/THD index consecutively [14].

## 5. Conclusion

At this stage, we propose TTPCL/THD >0.30 as a possible pathologic value because the mean TTPCL/THD index was >0.300 in all PI groups and ≤0.290 in all the controls. However, further prospective studies with larger study cohorts, a broader age distribution, and the inclusion of additional parameters like rotational analysis of the limb are needed to evaluate the significance of this novel index and its potential in clinical practice. The described TTPCL/THD index might circumvent the need for the impractical age- and gender-adjusted percentiles required when applying the TTPCL.

## Figures and Tables

**Table 1:** Patients' characteristics.

	PI	Control
*Adults*		
*n*	58	42
Age	28.7 years ± 1.8 SEM	21.2 years ± 1.1 SEM
Sex (male/female)	32/26	20/22
Side (right/left)	27/31	23/19

*Children*		
*n*	32	29
Age	12.3 years ± 0.4 SEM	13.3 years ± 0.3 SEM
Min/max	9/16 years	9/16 years
Sex (male/female)	22/10	16/13
Side (right/left)	13/19	15/14

**Table 2:** Measurements of the TTTG, TTPCL, THD, and TTPCL/THD for the control and patellar instability (PI) groups for adults and children.

	Adults			Children		
	Control	PI	*p* value	Control	PI	*p* value
TTTG (mm)	**9.3** ± 0.50	**13.4** ± 0.94	**<0.001**	**8.2** ± 0.55	**13.2** ± 1.10	**<0.001**
TTPCL (mm)	**20.2** ± 0.60	**22.1** ± 0.57	**0.031**	**20.6** ± 0.64	**22.6** ± 0.62	**0.031**
THD (mm)	**74.7** ± 0.82	**73.5** ± 0.82	0.247	**71.8** ± 1.03	**71.6** ± 1.00	0.902
TTPCL/THD	**0.270** ± 0.007	**0.301** ± 0.007	**0.005**	**0.288** ± 0.010	**0.316** ± 0.008	**0.033**

The results are expressed as the mean values of the group ± SEM.

**Table 3:** Gender-specific results for the TTTG, TTPCL, THD and TTPCL/THD in the healthy study population.

	Healthy adults			Healthy children		
	Male	Female	*p* value	Male	Female	*p* value
*n*	32	26		22	10	
TTTG (mm)	**9.7** ± 0.8	**8.8** ± 0.6	0.070	**8.7** ± 0.72	**7.3** ± 0.76	0.100
TTPCL (mm)	**22.1** ± 0.7	**17.9** ± 0.8	**<0.001**	**20.9** ± 0.76	**19.9** ± 1.20	0.562
THD (mm)	**79.1** ± 0.7	**69.3** ± 0.8	**<0.001**	**73.2** ± 1.32	**68.7** ± 1.16	**0.039**
TTPCL/THD	**0.279** ± 0.009	**0.259** ± 0.010	0.231	**0.287** ± 0.012	**0.290** ± 0.018	0.862

The results are expressed as the mean values of the group ± SEM.

## Data Availability

The full analytic data set will not be published in order to protect patients' rights and due to ongoing further studies but are available from the corresponding author on reasonable request.
